# PM_2.5_ Induces Pyroptosis via Activation of the ROS/NF-κB Signaling Pathway in Bronchial Epithelial Cells

**DOI:** 10.3390/medicina60091434

**Published:** 2024-09-02

**Authors:** Ji-Young Kang, Hyunsu Choi, Jeong-Min Oh, Minsu Kim, Dong-Chang Lee

**Affiliations:** 1 Division of Pulmonary, Allergy, and Critical Care Medicine, Department of Internal Medicine, Jeju National University Hospital, 15 Aran 13-gil, Jeju-si 63241, Republic of Korea; rkdwldud4221@gmail.com; 2 Clinical Research Institute, Daejeon St. Mary’s Hospital, Daeheung-dong, Jung-gu, Daejeon 34943, Republic of Korea; 20110201@cmcnu.or.kr (H.C.); yejmdh@cmcnu.or.kr (J.-M.O.); 3 Department of Otorhinolaryngology-Head and Neck Surgery, Daejeon St. Mary’s Hospital, College of Medicine, The Catholic University of Korea, 64 Daeheung-ro, Jung-gu, Daejeon 34943, Republic of Korea; tokimminsu@gmail.com

**Keywords:** particulate matter 2.5, reactive oxygen species, pyroptosis, respiratory epithelium, NF-kappa B

## Abstract

*Background and Objectives*: Fine particulate matter, PM_2.5_, is becoming a major threat to human health, particularly in terms of respiratory diseases. Pyroptosis is a recently discovered and distinct form of cell death, characterized by pore formation in the cell membrane and secretions of proinflammatory cytokines. There has been little research on the effect of PM_2.5_ on pyroptosis, especially in airway epithelium. We investigated whether PM_2.5_-related oxidative stress induces pyroptosis in bronchial epithelial cells and defined the underlying mechanisms. *Materials and Methods*: After exposure of a BEAS-2B cell line to PM_2.5_ concentration of 20 µg/mL, reactive oxygen species (ROS) levels, parameters related to pyroptosis, and NF-κB signaling were measured by Western blotting, immunofluorescence, and ELISA (Enzyme-linked immunosorbent assay). *Results*: PM_2.5_ induced pyroptotic cell death, accompanied by LDH (Lactate dehydrogenase) release and increased uptake of propidium iodide in a dose-dependent manner. PM_2.5_ activated the NLRP3-casp1-gasdermin D pathway, with resulting secretions of the proinflammatory cytokines IL-1β and IL-18. The pyroptosis activated by PM_2.5_ was alleviated significantly by NLRP3 inhibitor. In PM_2.5_-exposed BEAS-2B cells, levels of intracellular ROS and NF-κB p65 increased. ROS scavenger inhibited the expression of the NLRP3 inflammasome, and the NF-κB inhibitor attenuated pyroptotic cell death triggered by PM_2.5_ exposure, indicating that the ROS/NF-κB pathway is involved in PM_2.5_-induced pyroptosis. *Conclusions*: These findings show that PM_2.5_ exposure can cause cell injury by NLRP3-inflammasome-mediated pyroptosis by upregulating the ROS/NF-κB pathway in airway epithelium.

## 1. Introduction

Particulate matter (PM), a major air pollutant, is becoming a global public health concern. Defined by its aerodynamic diameter, PM_2.5_ (≤2.5 µm diameter)—often called fine particulate matter—is the particle size class with the most detrimental effects on the human body. PM_2.5_ has peculiar characteristics such as a large surface area; its adsorbed substances including polycyclic aromatic hydrocarbon, heavy metals, and microbes; and its easy transportation to the upper and lower respiratory tract as well as other systemic organs [1,2]. Cardiovascular diseases such as ischemic heart disease and stroke, respiratory diseases, lung cancer, type 2 diabetes mellitus, and preterm birth are the five most common conditions that can be attributed to PM_2.5_ exposure [3,4,5,6,7]. In 2019, there were an estimated 4.15 million ambient PM_2.5_-related deaths globally, a remarkable increase of 102% from 1990 to 2019 [8,9].

Previous research has shown that PM_2.5_-induced tissue damage primarily in the respiratory tract through oxidative stress, proinflammatory responses, cytotoxicity, apoptosis, and DNA damage [10,11,12,13,14]. In particular, reactive oxygen species (ROS), which are byproducts of oxidative stress, have adverse health effects on chronic obstructive lung disease (COPD), pulmonary fibrosis, and pulmonary infections through various mechanisms such as oxidization of DNA and lipids, TGF- β (transforming growth factor-beta) signaling activation, and epithelial barrier disruption [15,16,17]. We previously demonstrated that exposure to PM_2.5_ induces proinflammatory signaling activation and tight junction dysfunction via ROS generation in airway epithelial cell lines [14,18].

Pyroptosis is a newly discovered form of programmed cell death, which is executed by the gasdermin (GSDM) protein family and mediated by the inflammasome pathway [19]. Through pore formation in the cell membrane and release of the proinflammatory cytokines IL (interleukin)-1β and IL-18, pyroptosis not only eliminates harmful microbes from the body but also aggravates inflammatory conditions including respiratory diseases such as asthma or COPD [20]. Some research has shown that foreign insults such as allergens, viral infections, or cigarette smoke induce pyroptosis via the NLRP3 inflammasome signaling pathway in bronchial epithelial cells, resulting in persistent inflammation and/or airway remodeling [21,22,23]. However, few studies have examined the association between PM_2.5_ and pyroptosis, particularly in airway epithelium. This study aimed to investigate the effect of PM_2.5_ in human bronchial epithelial cells as a first-contact physiological barrier to noxious stimuli, as well as to elucidate the underlying mechanisms with a focus on NLRP3-inflammasome-induced pyroptosis with ROS involvement.

## 2. Materials and Methods

### 2.1. Chemical Reagents

Bronchial epithelial growth medium (BEGM) was obtained from Lonza (Walkersville, MD, USA). Antibiotic–antimycotic solution was purchased from Gibco (Thermo Fisher Scientific, Waltham, MA, USA). PM_2.5_ (NIST SRM 1650b), ROS scavenger N-acetylcysteine (NAC), NLRP3 Inhibitor MCC950, NF-κB inhibitor BAY 11-7082, and propidium iodide (PI) were purchased from Millipore Sigma (Saint Louis, MO, USA). The fluorescent stains 4,6-diamidino-2-phenylindole (DAPI) and 2,7-dichlorodihydrofluorescein diacetate (DCFH-DA) were obtained from Invitrogen (Carlsbad, CA, USA). The cell viability reagent, 3-(4,5-dimethylthiazol-2-yl)-2,5-dipehnlytetrazolium bromide, was from DoGen (Seoul, Korea). Antibodies against NOD-like receptor protein-3 (NLRP3), cleaved N-terminal gasdermin D (GSDMD-N), nuclear factor (NF)-κB p65, 3-glyceraldehyde phosphate dehydrogenase (GAPDH), and lamin-B1 were from Cell Signaling Technology (Danvers, MA, USA). The cleaved form of caspase-1 (Casp1) was from Santa Cruz Biotechnology (Santa Cruz, CA, USA).

### 2.2. Cell Culture and PM_2.5_ Exposure

The human bronchial epithelial cell line BEAS-2B was obtained from the American Type Culture Collection (Manassas, VA, USA). The cells were grown in culture dishes at 37 °C in 5% CO_2_ using BEGM medium containing all the recommended supplements (Lonza). The culture medium was replaced every two days. Cells were plated at 70–80% confluence and used the next day. A stock solution of PM_2.5_ at a concentration of 50 mg/mL was prepared in PBS and subsequently diluted to the desired concentrations, 10, 20, and 40 µg/mL in the culture medium.

### 2.3. Cell Viability Measurement and Lactate Dehydrogenase (LDH) Release Assay

After the cells were treated with PM_2.5_ at different concentrations, 0, 10, 20, and 40 μg/mL for 24 h, the culture medium was collected and centrifuged at 300 g for 5 min to precipitate suspended cells. The supernatants were stored at −80 °C until further analysis. Viability of the precipitated cells was measured after adding 3-(4,5-dimethylthiazol-2-yl)-2,5-diphenlytetrazolium bromide as a solution and incubating the cells for 2 h. Absorbance was recorded at 450 nm in a microplate reader (Bio-Rad, Hercules, CA, USA). As for cytotoxicity, LDH levels in the supernatants were determined using an LDH Cytotoxicity Assay Kit (DoGen) following the manufacturer’s protocol. Absorbances were read at 490 nm in the microplate reader.

### 2.4. Cell Death Assay

Cell death was measured by PI labeling. Cultured cells were stained with PI solution (2 µg/mL) in the dark at 37 °C for 30 min, observed under an inverted fluorescence microscope (Olympus IX73, Olympus, Tokyo, Japan) at 200× magnification, and the images were recorded.

### 2.5. Measurement of Intracellular ROS Levels

The DCFH-DA fluorescent dye probe was used to measure intracellular ROS production. After different treatments, cells were washed with PBS and incubated with 10 μM DCFH-DA in PBS at 37 °C for 20 min in the dark. The cells were washed twice with PBS, and fluorescent images were taken under a microscope at 200× magnification.

### 2.6. Extraction of Nuclear Protein

NF-κB p65 activity in the nuclear fractions was determined using a cell fractionation kit (Cell Signaling Technology) according to the manufacturer’s instructions. The detailed procedure has been explained previously [24]. Briefly, after a treatment, cells were washed twice with cold PBS and lysed with a cytosol extraction buffer. The supernatant was collected by centrifugation at a maximum speed for 5 min. Finally, the nuclear fraction was separated using a nuclear extraction buffer. The supernatant containing the nuclear protein extract was transferred to a fresh microcentrifuge tube and stored at −20 °C.

### 2.7. Western Blotting

Cells were washed twice with PBS and then lysed in a radioimmunoprecipitation assay lysis buffer (Elpis Biotech, Daejeon, Korea) containing protease inhibitor cocktail tablets (Roche Diagnostics, Mannheim, Germany); following this, they were centrifuged at 14,000× *g* for 15 min. Protein concentration was measured using the bicinchoninic acid kit (BCA Protein Assay Kit; Pierce, Rockford, IL, USA). A standardized quantity (20 µg) of protein extract was electrophoresed, immunoblotted, and detected as reported previously [24].

### 2.8. Immunofluorescence Assay

Cultured cells were seeded onto a coverslip at a density of 2 × 10^5^ cells/mL. After different treatments, the medium was discarded by draining, and the adhering cells were washed with PBS, fixed with 4% formaldehyde for 15 min, and permeabilized with 0.1% Triton X-100 for 10 min at room temperature. The cells were saturated with PBS containing 1% bovine serum albumin for 1 h at room temperature, then incubated with the relevant primary antibody (Cell Signaling Technology) at 1:100 dilution in PBS at 4 °C overnight. Next, cells were washed, stained with Alexa Fluor 488-conjugated goat anti-mouse antibodies (Invitrogen), and mounted with DAPI. The obtained images of the stained cells were measured quantitatively by ImageJ program (ImageJ software Version 1.54i, National Institute of Health, MD, USA). 

### 2.9. Quantification of IL-1β and IL-18 via an Enzyme-Linked Immunosorbent Assay (ELISA)

The protein expression levels of IL-1β and IL-18 in the culture supernatant were measured using BD OptEIA ELISA kits (BD Biosciences, San Jose, CA, USA). Values were expressed as pg/mL based on standard curves of recombinant cytokines.

### 2.10. Statistical Analysis

GraphPad Prism 5 (GraphPad Software Prism 5 Version 5.03, Inc., La Jolla, CA, USA) was used to analyze all the data. The significance of differences between control and experimental values was assessed using the unpaired *t*-test or one-way analysis of variance. All values are expressed as mean ± standard error of the mean (SEM).

## 3. Results

### 3.1. Exposure to PM_2.5_ Induced Cell Death in BEAS-2B Cells in a Dose-Dependent Manner

**Figure 1:**
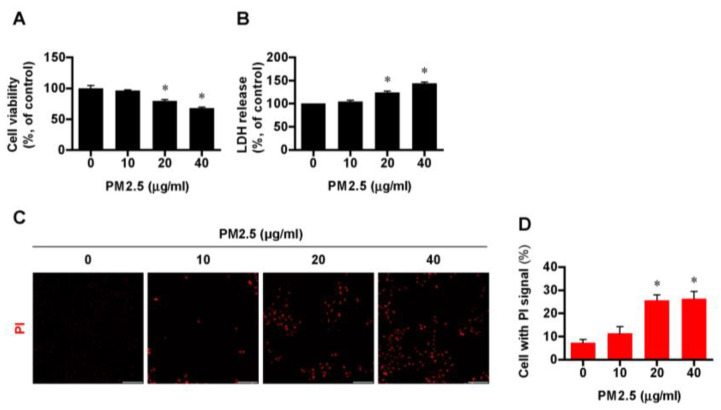
PM_2.5_−induced cell death in BEAS−2B cells. Cells were treated with PM_2.5_ at different concentrations (0, 10, 20, and 40 µg/mL) for 24 h. (**A**,**B**) Effects of PM_2.5_ on the cell viability and the amount of LDH release in BEAS-2B cells. (**C**,**D**) Cell death assessed by PI uptake (red fluorescence) and the ratio of PI-positive cells using ImageJ software. All experiments were performed in triplicate. Values are the mean ± SEM. *, *p* < 0.05 compared with control; scale bar = 100 µm; LDH, lactate dehydrogenase; PI, propidium Iodide.

To explore the effect of PM_2.5_ on cell death, BEAS-2B cell lines were treated with varying concentrations of PM_2.5_ (0, 10, 20, and 40 µg/mL) for 24 h, in reference to previous studies [14,25]. As PM_2.5_ directly affects in the airway and lungs, we selected the BEAS-2B cell lines, human bronchial epithelial cells. Cell viability in bronchial epithelial cells decreased significantly (*p* < 0.05) from a PM_2.5_ concentration of 20 µg/mL, compared with the control group of no exposure to PM_2.5_ (Figure 1A). As for cytotoxicity, Figure 1B shows that more LDH was released with an increasing concentration of PM_2.5_. Cells with a PI signal, a surrogate marker of cell death, were more pronounced fluorescence uptake in a dose-dependent manner as determined by quantified fluorescence uptake (Figure 1C,D). Based on these results, 20 µg/mL of PM_2.5_, which induced a marked inflammatory response with an acceptable cytotoxicity, was chosen for further experiments with various inhibitors.

### 3.2. PM_2.5_ Involved Activation of the NLRP3 Inflammasome and Release of IL-1β and IL-18 in BEAS-2B Cells

**Figure 2:**
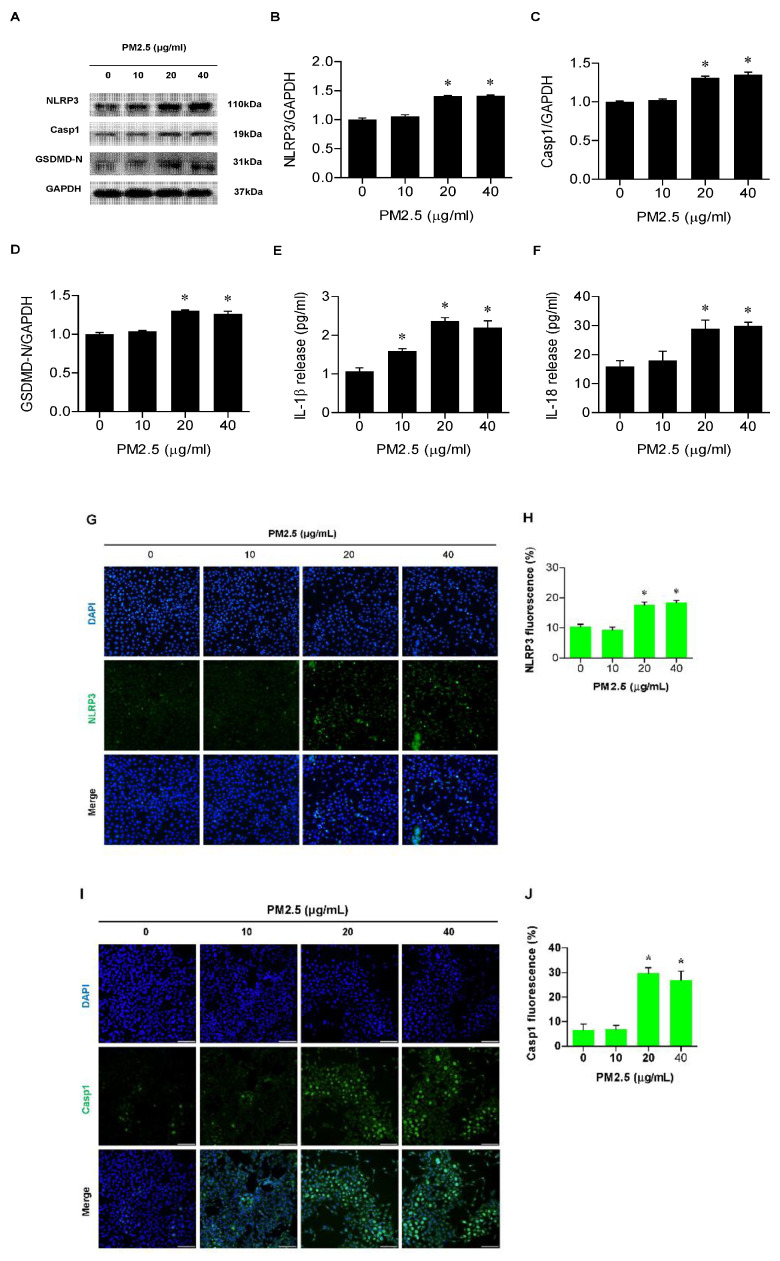
PM_2.5_−induced activation of the NLRP3 inflammasome and release of IL−1β and IL−18 in BEAS−2B cells. Cells were treated with PM_2.5_ at different concentrations (0, 10, 20, and 40 µg/mL) for 24 h. (**A**–**D**) Protein levels and densitometric analyses of NLRP3, Casp1, and GSDMD-N in cells. (**E**,**F**) Concentrations of IL−1β and IL−18 by ELISA. (**G**,**H**) NLRP3 detection by immunofluorescent staining and quantitation of the fluorescent signal. (**I**,**J**) Casp1 expression by immunofluorescent staining and quantification of its fluorescence intensity. All experiments were performed in triplicate. Values are the mean ± SEM. *, *p* < 0.05 compared with the control; scale bar = 100 μm.

To determine whether the NLRP3 inflammasome is activated in BEAS-2B cells after PM_2.5_ treatment, we measured the expression of NLRP3 inflammasome proteins, NLRP3, Casp1, and GSDMD-N. In Western blot analysis, the expression of NLRP3, Casp1, and GSMDM-N increased markedly with PM_2.5_ treatment, especially at 20 and 40 µg/mL (Figure 2A–D). The levels of proinflammatory cytokines associated with pyroptosis, IL-1β, and IL-18 were significantly higher than in the control after PM_2.5_ exposure (Figure 2E,F). In the immunofluorescence assay of NLRP3 and Casp1, the fluorescent intensity of the two vital mediators involved in pyroptosis became stronger as exposure to PM_2.5_ increased (Figure 2G–J).

### 3.3. PM_2.5_ Exposure Triggered Pyroptosis in BEAS-2B Cells in a NLRP3-Inflammasome-Dependent Manner

**Figure 3:**
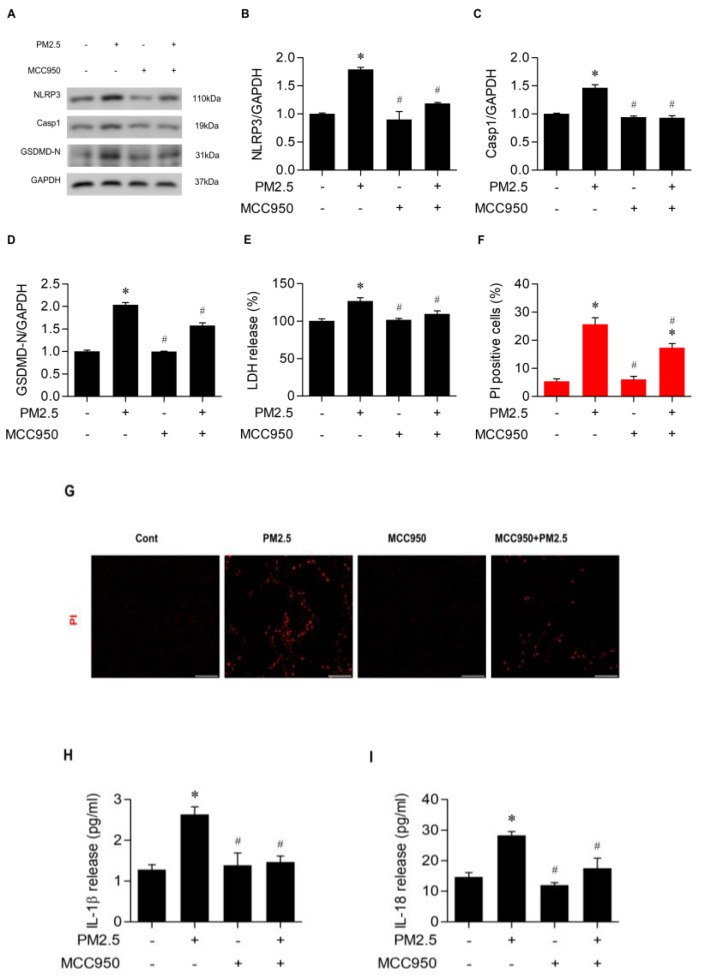

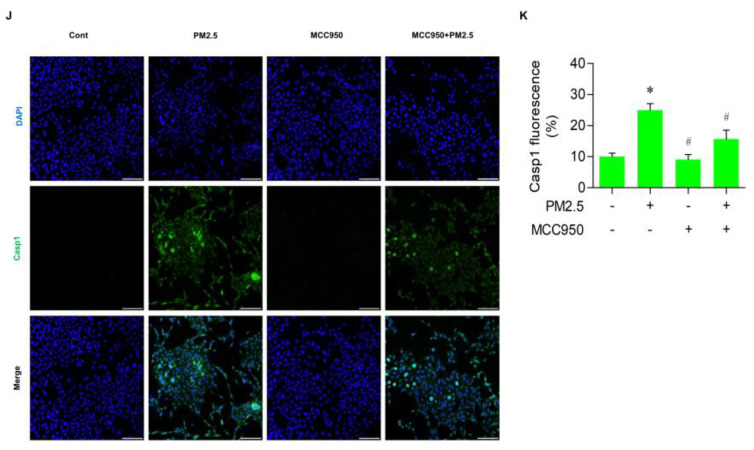
PM_2.5_ triggered pyroptosis of BEAS−2B cells in an NLRP3−inflammasome−dependent manner. Cells were treated with PM_2.5_ of 20 µg/mL for 24 h after treatment with MCC950, a NLRP3 inflammasome inhibitor (0.5 µM) for 2 h. (**A**–**D**) Western blotting results and quantitation analysis for NLRP3, Casp1, and GSDMD−N expression in BEAS−2B cells treated with MCC950 and exposed to PM_2.5_ or not. (**E**) Effects of MCC950 on the amount of LDH release in PM_2.5_-treated cells. (**F**,**G**) Effects of MCC950 on PM_2.5_-induced PI staining in BEAS-2B cells. (**H**,**I**) Effect of MCC950 on IL−1β and IL−18 production in PM_2.5_-treated cells. (**J**,**K**) Effect of MCC950 on Casp1 expression measured by immunofluorescent staining and quantification of its fluorescence intensity. All experiments were performed in triplicate. Values are the mean ± SEM. *, *p* < 0.05 compared with the control; #, *p* < 0.05 compared with the PM_2.5_−treated control; scale bar = 100 µm. LDH, lactate dehydrogenase; PI, propidium Iodide.

To further confirm an effect of the NLRP3 inflammasome on pyroptosis induced by PM_2.5_ in airway epithelial cells, BEAS-2B cells were treated for 2 h with MCC950, an NLRP3 inflammasome inhibitor, followed by a 24 h exposure to PM_2.5_ of 20 µg/mL. The expressions of pyrotosis parameters such as NLRP3, Casp1, and GSDMD-N were significantly reduced by prior MCC950 administration with PM_2.5_ exposure, compared to the PM_2.5_-treated control (Figure 3A–D). Moreover, LDH release and cells with positive PI staining decreased with MCC950 treatment and subsequent PM_2.5_ exposure (Figure 3E–G). MCC950 also diminished the concentration of IL-1β and IL-18 (Figure 3H,I). Taken together, these results confirm that PM_2.5_-induced pyroptosis in BEAS-2B cells is NLRP3-inflammasome-dependent.

### 3.4. PM_2.5_ Activated the ROS/NF-ĸB Pathway in BEAS-2B Cells

**Figure 4:**
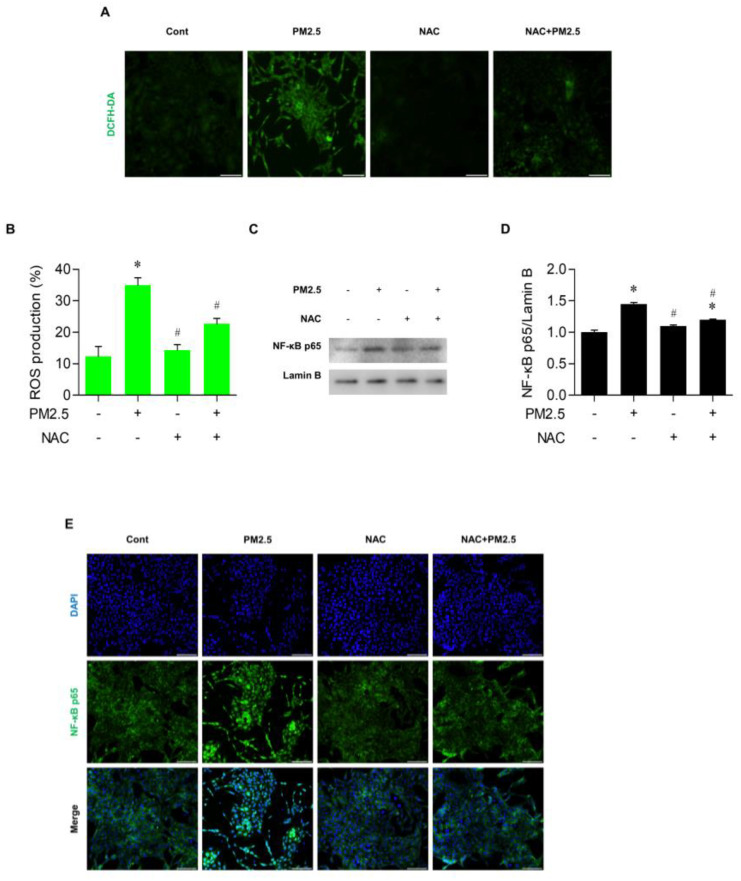
PM_2.5_ activated the ROS/NF−κB pathway in BEAS−2B cells. Cells were treated with 5 mM NAC, a ROS scavenger, for 1 h, and then were treated with 20 µg/mL PM_2.5_ for 24 h. (**A**) The intracellular ROS levels shown by DCFH-DA fluorescence. (**B**) Relative intensity of ROS production in DCFH−DA fluorescence. (**C**) Expression of NF-κB p65 and lamin−B1 by Western blotting. (**D**) Quantitation of the relative density of NF-κB p65 to lamin−B1. (**E**) Nuclear localization of NF−κB p65 by immunofluorescence staining. All experiments were performed in triplicate. Values are the mean ± SEM. *, *p* < 0.05 compared with the control; #, *p* < 0.05 compared with the PM_2.5_-treated control; scale bar = 50 µm.

Consistent with results of our previous studies [14,18], PM_2.5_ at 20 μg/mL markedly increased intracellular ROS production in bronchial epithelial cells. However, prior treatment with 5 mM NAC, a ROS scavenger, for 1 h before the exposure to PM_2.5_ significantly decreased the ROS level in cells compared with the NAC-untreated and PM_2.5_-exposed control (*p* < 0.05) in the DCFH-DA fluorescence assay (Figure 4A,B). To investigate whether the increased ROS level participates in the activation of NF-κB in PM_2.5_-treated cells, the expression of NF-κB p65 protein in cells treated with both NAC and PM_2.5_ was examined by Western blotting. As shown in Figure 4C–E, PM_2.5_ induced nuclear NF-κB p65 expression and increased fluorescence, but the effect was reversed by NAC administration. Therefore, we conclude that PM_2.5_ activates the NF-κB signaling pathway by increasing the intracellular ROS level in BEAS-2B cells.

### 3.5. NAC Inhibited the Expression of NLRP3 and Casp1 in PM_2.5_-Treated BEAS-2B Cells

**Figure 5:**
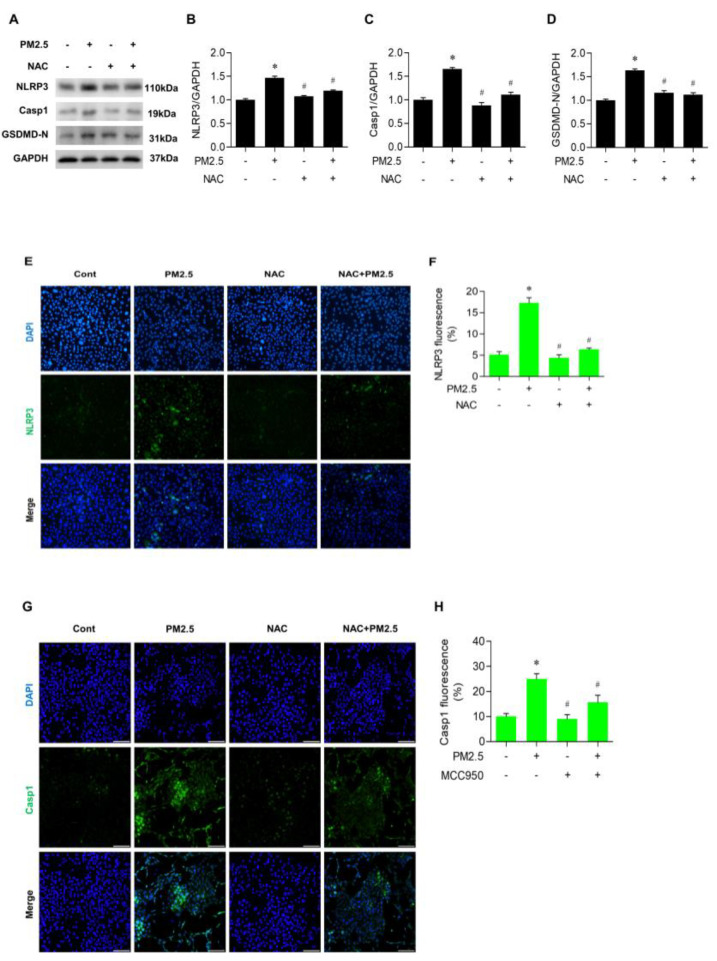
NAC inhibited the expression of NLRP3 and Casp1 in PM_2.5_−treated BEAS−2B cells. Cells were treated with 5 mM NAC for 1 h and then administered with 20 µg/mL PM_2.5_ for 24 h. (**A**–**D**) Protein levels and densitometric analyses of NLRP3, Casp1, and GSDMD−N in cells. (**E**,**F**) NLRP3 measured by immunofluorescent staining and quantified by the fluorescent signal. (**G**,**H**) Casp1 detection by immunofluorescent staining and its quantitation of the fluorescence intensity. All experiments were performed in triplicate. Values are the mean ± SEM. *, *p* < 0.05 compared with the control; #, *p* < 0.05 compared with the PM_2.5_-treated control; scale bar = 100 µm.

We evaluated whether increased ROS by PM_2.5_ is associated with the activation of the NLRP3 inflammasome. Figure 5A–D shows that pretreatment with NAC before PM_2.5_ exposure markedly decreased the level of NLRP3, Casp1, and GSDMD-N in BEAS-2B cells (*p* < 0.05). In the immunofluorescence method, the expression of NLRP3 was reduced significantly by NAC in PM_2.5_-treated cells (Figure 5E,F). In addition, the change of Casp1 expression showed a similar pattern (Figure 5G,H). These findings support the notion that increased ROS generated by PM_2.5_ is important for inducing NLRP3-inflammasome-mediated pyroptosis.

### 3.6. BAY Diminished Pyroptotic Cell Death in PM_2.5_-Treated BEAS-2B Cells

**Figure 6:**
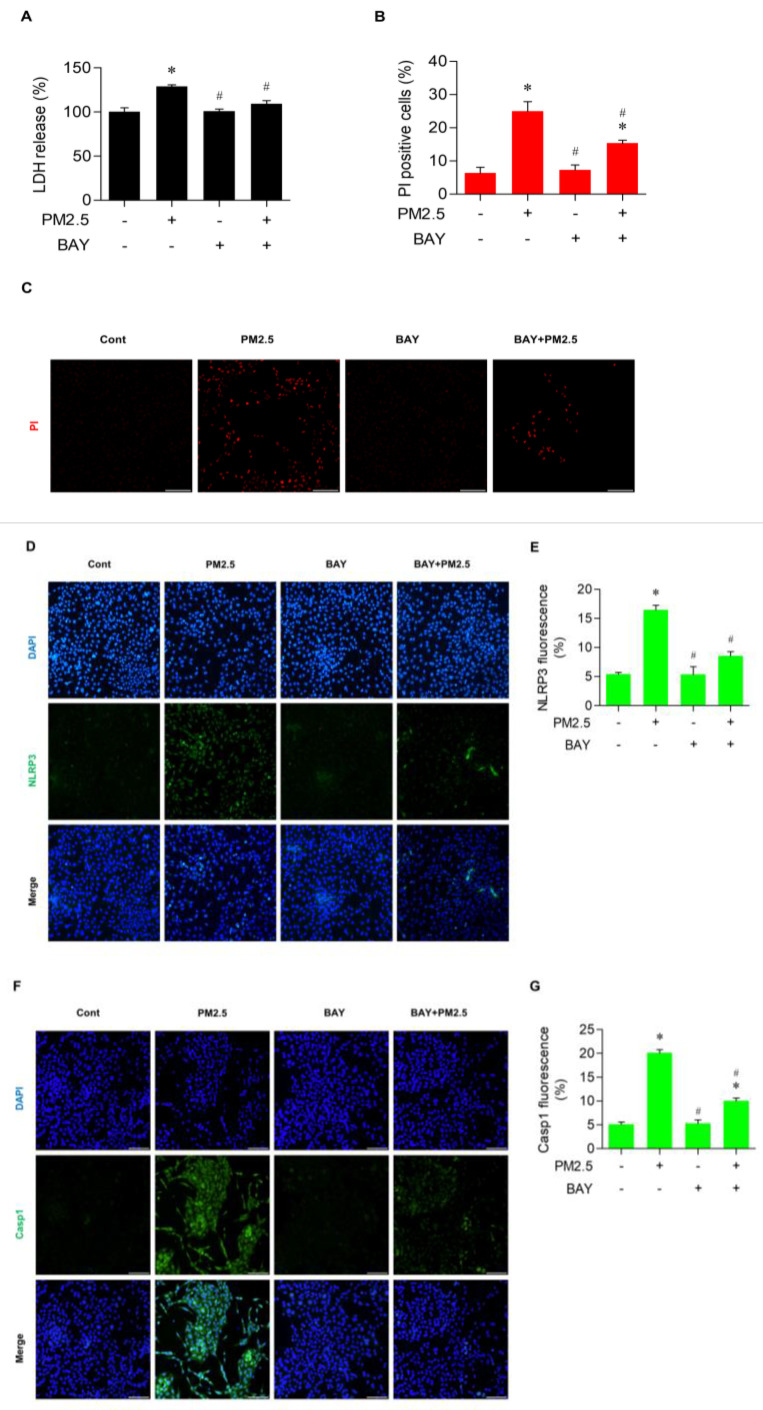
BAY, a NF−κB inhibitor, alleviated pyroptotic cell death in PM_2.5_−treated BEAS−2B cells. Cells were treated with 10 µM BAY for 30 min, followed by 20 µg/mL PM_2.5_ for 24 h. (**A**) Cytotoxicity as measured by the amount of LDH release. (**B**,**C**) PI−stained cell death with the ratio of PI−positive cells quantified by ImageJ software. (**D**,**E**) Effect of BAY on NLRP3 expression detected in immunofluorescence and the quantification of its fluorescence. (**F**,**G**) Effect of BAY on Casp1 measured by immunofluorescent staining and its fluorescent intensity. For all experiments, triplicated data are shown as mean ± SEM. *, *p* < 0.05 compared with the control; #, *p* < 0.05 compared with the PM_2.5_-treated control; scale bar = 100 µm.

To further clarify the involvement of NF-ĸB signaling in the effect of ROS on pyroptosis caused by PM_2.5_ exposure, cells were treated 10 µM BAY, an NF-κB inhibitor, for 30 min before exposure to PM_2.5_ at 20 µg/mL for 24 h. As shown in Figure 6A, the cytotoxicity as measured by LDH level decreased significantly in cells previously treated with BAY compared with activity in cells exposed to PM_2.5_ only. In addition, BAY significantly decreased the proportion of PI-positive cells caused by PM_2.5_ exposure (Figure 6B,C). The expression of NLRP3 and Casp1, the crucial players in pyroptosis, were lowered considerably by BAY treatment in the immunofluorescence assay with PM_2.5_ exposure (Figure 6D,G). Collectively, these results provide strong evidence that the activation of NF-κB signaling pathway is involved in PM_2.5_-induced cell pyroptosis in BEAS-2B cells.

## 4. Discussion

In this study, we demonstrated that PM_2.5_ exposure induces pyroptosis and production of the proinflammatory cytokines IL-1β and IL-18 via the NLRP3 inflammasome in bronchial epithelial cells and that the effect is modulated by activating the ROS/NF-κB signaling pathway.

PM_2.5_ causes cytotoxicities by regulating ROS-induced cell death pathways such as apoptosis, autophagy, and pyroptosis. Newly discovered cell death pathways such as ferropotosis by iron-dependent lipid peroxidation and necroptosis, a form of caspase-independent programmed necrosis, could also be affected by PM_2.5_ via ROS stimuli [26]. Various factors such as differences in dose or duration of PM_2.5_ exposure, the nature of components in PM_2.5_, and the types of cells or organs involved could affect the specific profile of cell death pathways. As for pyroptosis, the best-known involved process is a classical or canonical inflammasome-induced pathway mediated by Casp1 and executed by GSDMD [27]. We also verified that PM_2.5_ exposure causes pyroptotic cell death in a dose-dependent manner by ROS generation through the NLRP3-inflammasome-induced classical pathway as shown by our step-by-step experiments with the ROS scavenger NAC and the NLRP3 inhibitor MCC950.

The airway epithelium is a primary line of defense against exogenous insults in the respiratory tract. It plays a critical role as a physical barrier as well as in inflammation and innate immune responses. Previous in vitro research on pyroptosis in PM_2.5_ exposure has focused on alveolar macrophages, the main inflammatory cells in the lung [28,29]. There have been few studies of the effect of PM_2.5_ on pyroptosis in airway epithelial cells. In the present study, we have confirmed that the ROS–NLRP3 inflammasome–Casp1–GSDMD axis is activated in bronchial epithelial cells exposed to PM_2.5_. Based on the fact that defects in the airway epithelium are closely associated with asthma, COPD, cystic fibrosis, and infection [30], the bronchial epithelial cell injury caused by pyroptosis after PM exposure could have an impact on the pathogenesis of various respiratory diseases. Interestingly, Ren et al. reported that pathways other than pyroptosis, such as apoptosis or cell cycle arrest, occur via upregulating ROS after PM_2.5_ exposure and lead to cell damage in a macrophage cell line [29]. Further research is needed to explore what the most dominant pathways in the various cytotoxicities are and how they interact with different PM_2.5_ exposures.

This study determined that the NF-κB signaling pathway participates in pyroptotic cell death by ROS regulation in bronchial epithelial cells after PM_2.5_ treatment; an experiment with NF-ĸB inhibitor showed reduced expression of Casp1, with decreased LDH concentrations and fewer PI positive cells. NF-κB is a key transcriptional factor with a critical role in inflammation and cell death pathways. Because an activated NF-κB signal induces NLRP3 inflammasome expression and production of proinflammatory cytokines such as IL-1β [31,32], it could trigger and maintain pyroptosis. Some evidence shows that ROS release by PM_2.5_ exposure activates NF-ĸB signaling and enhances a downstream inflammatory cascade in airway epithelial cells [31,33]. Further, Song et al. demonstrated that regulation of mir-331 expression by PM_2.5_ exposure with ROS generation maintains NF-ĸB signaling activation in human airway epithelium [31]. In addition, PM_2.5_-induced apoptosis or autophagy also involve the NF-ĸB pathway [26]. Dou et al. reported that cooking-oil-fume-derived PM_2.5_ activates the MAPK/NF-ĸB/STAT1 pathway and results in inflammation as well as apoptosis and cell damage [34]. IL-1β is one of the major proinflammatory cytokines, produced in a proactive form by some types of immune cells such as macrophages or dendritic cells. It exerts biologic effects after transformation to its active form and extracellular secretion through pyroptosis. It increases pulmonary inflammation and fibrosis by inducing neutrophil and macrophage infiltrations and disruption of elastic fiber in alveoli, with increased matrix metalloproteinases, MMP-9 and MMP-12 [35]. Additionally, IL-1β has a substantial role in lung cancer development via regulation of tumor growth, invasiveness, and angiogenesis, which were demonstrated in a clinical study using the anti-IL-1β antibody canakinumab [36]. Unlike IL-1β, IL-18 is produced in various cell types, but like IL-1β it is stored as a proactive form to be activated by Casp1 in the NLRP3 inflammasome. IL-18 has pleotropic functions depending on cell type and the cytokine milieu, such as production of interferon-γ (IFN-γ) as well as the other cytokines IL-4 and IL-13. In an animal model study by Sugimoto et al., IL-18 with antigen activated Th1 cells with the production of IFN-γ, IL-19, and IL-13, leading to severe asthma development [37]. We observed that IL-1β and IL-18 were released by PM_2.5_ exposure in an NLRP3-inflammasome-dependent manner in bronchial epithelial cell lines, which could induce lung injury.

There are some weaknesses to be addressed in this work. We conducted an in vitro study using the bronchial epithelial cell line and did not verify in vitro experiments, which could limit the generalizability of our findings. In addition, we focused mainly on the ROS/NF-κB signal pathway in the PM_2.5_-induced pyroptosis. A recent study showed that hexavalent chromium, one of the major components in PM_2.5_, induces inflammasome-mediated pyroptosis in airway epithelial cells [38]. Further research on this topic is needed for a better understanding of PM_2.5_-related toxicity and target development to reverse its harmful effects. Lastly, we chose the concentration of PM_2.5_ based on the results of cell cytotoxicity and viability. However, the exposed level of PM_2.5_ in this experimental setting might not be similar to it in a real-world situation. This could be another limitation in the interpretation of our findings. Various diseases such as multiple sclerosis, inflammatory bowel diseases, autoimmune thyroiditis, anti-synthetase syndrome, as well as myocardial infarction, are associated with NLRP3 inflammasome [39,40,41]. As we revealed that NLRP3 inflammasome is activated and induces pyroptotic cell injury via ROS/NF-κB signal by PM_2.5_ exposure, targeting mediators involved in this cascade would be a potential therapeutic option for those intractable diseases.

In conclusion, this study has shown that PM_2.5_ induces pyroptosis in airway epithelial cells via the ROS–NLRP3 inflammasome–Casp1–GSDMD axis with NF-κB signaling activation. Considering that pyroptosis might be involved in various respiratory diseases such as asthma, COPD, and lung cancer and that PM_2.5_ is one of the most important factors in those diseases’ pathogenesis, these findings provide valuable clues to explore new therapeutic candidates in pulmonary diseases aggravated by PM_2.5_.

## Data Availability

Data will be available from the corresponding author on reasonable request.

## Abbreviations

BEGM: bronchial epithelial growth medium; Casp 1, caspase-1; COPD, chronic obstructive pulmonary disease; ELISA, enzyme-linked immunosorbent assay; GSDM, gasdermin; GSDMD-N, N-terminal gasdermin D; IL, interleukin; LDH, lactate dehydrogenase; NAC, N-acetylcysteine; NF-κB: nuclear factor-κB; NLRP3, NOD-like receptor protein-3; PI, propidium iodide; PM, particulate matter; ROS, reactive oxygen species.
